# Impact of Methylated Cyclodextrin KLEPTOSE^®^ CRYSMEB on Inflammatory Responses in Human In Vitro Models

**DOI:** 10.3390/ijms25179748

**Published:** 2024-09-09

**Authors:** Damien Truffin, Flora Marchand, Mathias Chatelais, Gérald Chêne, Laure Saias, Frauke Herbst, Justin Lipner, Alastair J. King

**Affiliations:** 1 Roquette Frères, Rue de la Haute Loge, 62136 Lestrem, France; 2 ProfileHIT, 7 Rue du Buisson, 44680 Sainte-Pazanne, France; 3 Ambiotis, 3 Can Biotech 3 r Satellites, 31400 Toulouse, France; 4 Eurofins Discovery, 6 Research Park Drive, St. Charles, MO 63304, USA

**Keywords:** methylated cyclodextrin, cyclodextrin, KLEPTOSE^®^ CRYSMEB, anti-inflammatory, immunity, HUVEC, BCS, T cell, Diversity PLUS^®^ panel, chemokines, polyunsaturated fatty acid, lipoxins, resolvins, maresins, protectins, peripheral blood mononuclear cell, screening, cytokine

## Abstract

KLEPTOSE^®^ CRYSMEB methylated cyclodextrin derivative displays less methylated group substitution than randomly methylated cyclodextrin. It has demonstrated an impact on atherosclerosis and neurological diseases, linked in part to cholesterol complexation and immune response, however, its impact on inflammatory cascade pathways is not clear. Thus, the impact of KLEPTOSE^®^ CRYSMEB on various pharmacological targets was assessed using human umbilical vein endothelial cells under physiological and inflammatory conditions, followed by screening against twelve human primary cell-based systems designed to model complex human tissue and disease biology of the vasculature, skin, lung, and inflammatory tissues using the BioMAP^®^ Diversity PLUS^®^ panel. Finally, its anti-inflammatory mechanism was investigated on peripheral blood mononuclear cells to evaluate anti-inflammatory or pro-resolving properties. The results showed that KLEPTOSE^®^ CRYSMEB can modulate the immune system in vitro and potentially manage vascular issues by stimulating the expression of molecules involved in the crosstalk between immune cells and other cell types. It showed anti-inflammatory effects that were driven by the inhibition of pro-inflammatory cytokine secretion and could have different impacts on different tissue types. Moreover, this cyclodextrin showed no clear impact on pro-resolving lipid mediators. Additionally, it appeared that the mechanism of action of KLEPTOSE^®^ CRYSMEB seems to not be shared by other well-known anti-inflammatory molecules. Finally, KLEPTOSE^®^ CRYSMEB may have an anti-inflammatory impact, which could be due to its effect on receptors such as TLR or direct complexation with LPS or PGE2, and conversely, this methylated cyclodextrin could stimulate a pro-inflammatory response involving lipid mediators and on proteins involved in communication with immune cells, probably via interaction with membrane cholesterol.

## 1. Introduction

Cyclodextrins (CDs) are cyclic glucose chain oligomers that are composed of different numbers of α-1-4-linked glucose residues. They contain a cone-like cavity into which compounds like active ingredients, cholesterol, and derivatives may enter to form a water-soluble complex [1]. Cyclodextrins derived from native β-cyclodextrins (β-CD) show different biological and physical properties, mainly depending on their degree of substitution. Among these cyclodextrin derivatives, one particular methylated cyclodextrin differs from the randomly methylated β-CD (RAMEB): KLEPTOSE^®^ CRYSMEB, which displays fewer methylated group substitutions than RAMEB.

Native cyclodextrins exhibit poor solubility in water; cholesterol–cyclodextrin complexes also exhibit poor solubility in water compared to derivative cyclodextrins, especially methylated cyclodextrins [2]. Nevertheless, low-substituted methylated derivatives such as KLEPTOSE^®^ CRYSMEB show weaker interactions with cholesterol than dimethylcyclodextrin, trimethylcyclodextrin, or RAMEB [2]. However, this lower impact on cholesterol extraction and solubilization is correlated with a lower impact on cellular membranes, inducing better cell viability than other methylated cyclodextrins with a cytotoxicity value between hydroxypropyl β-cyclodextrin (HPβ-CD) and β-CD [3]. This makes this molecule suitable for complexation with cholesterol and oxysterol, which are involved in various disease phenotypes [4], with a better safety profile than RAMEB and other methylated cyclodextrins, especially for intravenous routes of administration.

Atherosclerosis is an inflammatory disease that is linked to high blood concentrations of small dense low-density lipoprotein cholesterol (sdLDL-C) [5]. Therapies using cyclodextrin (e.g., KLEPTOSE^®^ CRYSMEB) as a cholesterol-sequestrating agent have demonstrated an impact on atherosclerosis [6]. Moreover, the inflammatory response in atherosclerosis was investigated with KLEPTOSE^®^ CRYSMEB, where the authors demonstrated that T lymphocyte content was reduced in atherosclerotic plaques, with the mode of action being immunomodulation and Th1 polarization [7]. In addition, other cyclodextrins such as HPβ-CD were able to inhibit atherosclerosis, not only by increasing the efflux of cholesterol but by additionally involving anti-inflammatory mechanisms [8]. They also induced the regression of atherosclerosis via macrophage reprogramming to improve cholesterol efflux and exert anti-inflammatory effects [9]. In addition, HPβ-CD and α-CD inhibited cholesterol crystal formation and the activation of complement on monocytes, and HPβ-CD reduced immunoglobulin G (IgG) deposition [10,11]. This anti-inflammatory effect of CDs is linked to a specific effect against cholesterol crystals [8]. Moreover, CDs may potentially be useful as active agents for the treatment of neurological disorders: first for diseases involving cholesterol depletion such as Niemann Pick type C disease, and second, as anti-aggregative compounds for amyloid-related disorders such as Alzheimer’s disease or α-synuclein in Parkinson’s disease [12]. However, this has yet to be fully evaluated as clinical testing for the treatment of Alzheimer’s disease is ongoing. All the effects induced by CDs were linked to cholesterol complexation. Nevertheless, the potential effect of these compounds and their potential impacts on cellular metabolism, interactions between cells from different tissues, and pro- and anti-inflammatory cascade pathways is not clear.

Regarding the impact of cyclodextrins on different pathologies that could involve inflammatory mechanisms, the aim of this study was to investigate, using in vitro models, the impact of methylated cyclodextrin KLEPTOSE^®^ CRYSMEB on the immune system and especially the crosstalk between immune cells and cytokine secretion.

In the present study, we assessed the impact of KLEPTOSE^®^ CRYSMEB on various pharmacological targets using human umbilical vein endothelial cells (HUVECs) in physiologically normal and inflammatory conditions, followed by screening against a panel of twelve human primary cell-based systems designed to model complex human tissue and disease biology of the vasculature, skin, lung, and inflammatory tissues using the BioMAP^®^ Diversity PLUS^®^ panel. Finally, the anti-inflammatory mechanism of KLEPTOSE^®^ CRYSMEB was investigated on peripheral blood mononuclear cells (PBMCs) to evaluate anti-inflammatory or pro-resolving properties. HUVEC was a model of choice to study immunomodulation in humans. Indeed, located at the blood/organ interplay, endothelial cells act as a first-line defense system to enhance/decrease immune responses by (i) modulating chemokine secretion, co-stimulatory and co-inhibitory receptors, leucocyte recruitment, and phagocytic properties [13], and (ii) presenting exogenous peptides through HLA-DR (human leukocyte antigen DR) in the IFN-γ context [14]. 

As some targets can be modulated differently depending on the stimulation used [15], we investigated surface expression with different stimulations to get the strongest expression of each target, for example, HLA-DR is not expressed in a TNF-α context. In this method, cells were treated in the presence (“inflammatory condition”) or absence (“non-inflammatory condition”) of TNF-α or IFN-γ. The HUVECs were thus considered a relevant model, which was used to specifically evaluate the effects of KLEPTOSE^®^ CRYSMEB in a first-line immunomodulation screening strategy. We focused on the major; the expression of molecules that are involved in the crosstalk between immune cells and surrounding cells like endothelial cells: ICAM-1 (Intercellular Adhesion Molecule 1) and GITRL (Glucocorticoid-induced TNFR-related protein Ligand), which are involved in transmigration [16,17] and atherosclecortic plaque stabilization [18,19]; HLA-E (HLA class I histocompatibility antigen, alpha chain E); HLA-1/HLA-ABC/MHC Class I (human leukocyte antigen A, B, C, or MHC class I A, B, C); HLA-2/HLA-DR/MHC Class II (human leukocyte antigen DR or MHC class II DR), which are involved in antigenic presentation to both innate and adaptative immune cells [15,20,21]; CD55 (Complement decay-accelerating factor); CD59 (CD59 glycoprotein); CD39 (Ectonucleoside triphosphate diphosphohydrolase-1), which contributes to vascular protection [22,23,24]; HVEM (Herpesvirus entry mediator); IFN-γR1 (Interferon gamma receptor 1); and TNFR1 (Interferon gamma receptor 1), which are involved in the initiation of inflammatory response in cells [25,26,27].

Human primary cell-based models within the BioMAP^®^ Diversity PLUS^®^ panel represent a broad set of tissue and disease biology, including vascular biology, adaptive and innate immune responses, respiratory biology, fibrosis, and skin-relevant biology [28,29]. The BioMAP^®^ systems used in this study are constructed with one or more primary cell types pooled from three or more healthy human donors, with stimuli (such as cytokines or growth factors) added to stimulate relevant signaling networks that naturally occur in human tissues or under pathological conditions.

BioMAP^®^ profiling creates a unique signature of changes in protein biomarker readouts when normalized to vehicle controls within individual system environments. Biomarker readouts (7–17 per system) are therapeutically and biologically relevant, are predictive of disease outcomes or specific drug effects, and have been validated using agents with known mechanisms of action [29,30,31]. Details of these methods have been published previously by Shah et al. [28]. The BioMAP^®^ Diversity PLUS^®^ panel comprises twelve cell systems and measures 148 total biomarker readouts to probe the biological impacts of test agents in a broad range of human tissue and disease biology. This panel measures a diverse range of readouts, including cell surface receptors, cytokines, chemokines, matrix molecules, and enzymes. Levels of these biomarkers are measured quantitatively by immune-based methods that detect proteins, including ELISA, as well as functional assays that measure cell proliferation and viability.

Finally, the KLEPTOSE^®^ CRYSMEB anti-inflammatory mechanism was assessed in a PBMC culture model to evaluate anti-inflammatory responses following cytokine secretion, pro-inflammatory mediators (Prostaglandins, Thromboxanes, Leukotrienes), or Specialized Pro-resolving Mediators (SPMs). These SPMs originate from omega-6 and omega-3 polyunsaturated fatty acid (PUFA) metabolism and are classified into four main families: Lipoxins, Resolvins, Maresins, and Protectins [32,33]. The simulation of inflammation was performed using LPS for the Toll-like receptor route for cytokine expression and phorbol 12-myristate 13-acetate (PMA) and an antibiotic (Calimycin, calcium route activator) for pro-inflammatory mediators involving cyclooxygenase 2 (COX2) as well as the lipoxygenase pathways (5-LOX, 12-LOX, and 15-LOX).

## 2. Results

### 2.1. Results of HUVEC Immuno-Profiling

**Figure 1:**
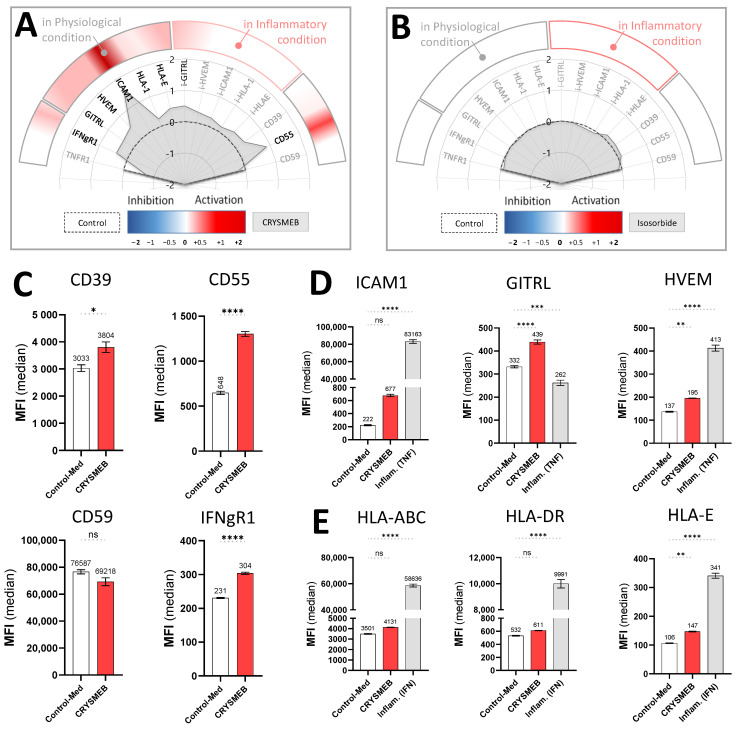
Immuno-profiling of HUVECs following treatment with KLEPTOSE^®^ CRYSMEB and ISOSORBIDE C PHARMA under physiologically normal (**A**) and inflammatory (**B**) conditions. Representative immunostimulatory protein responses, following 48 hours of treatment with the inflammatory cytokines TNF-α and IFN-γ (**C**). Effect of KLEPTOSE^®^ CRYSMEB on antigen presentation, cell recruitment (**D**), immunomodulation of co-stimulatory proteins and vasculo-protective markers (**E**). Control-Med (medium alone), Control-TNF (TNF-α alone), and Control-IFN (IFN-γ alone). Results are expressed as mean ± SEM, unpaired *t*-test, two-tailed for C and one-way ANOVA followed by Tukey’s multiple comparison test (**D**,**E**); ns: non-significant, *p*-value: **** *p* < 0.0001, *** *p* < 0.001, ** *p* < 0.01, * *p* < 0.05.

In vitro HUVEC profiling revealed that ISOSORBIDE C PHARMA showed no impact on biomarker modulation under physiologically normal or inflammatory conditions. As a negative control, this demonstrated that the model was valid since isosorbide is known to have no pharmacological effects [34] (Figure 1B). Moreover, the immunostimulation of HUVECs alone following 48 hours of treatment with the inflammatory cytokines TNF-α and IFN-γ, showed a marked increase in the immune-related markers HVEM, ICAM-1, HLA-1, HLA-E, and HLA-DR, demonstrating that the model was valid for the inflammatory conditions tested and for measuring HUVEC responses. KLEPTOSE^®^ CRYSMEB elicited statistically significant modulation of key biomarkers towards increased levels of expression of some of the immune-related markers, such as IFN-γR1, ICAM-1, HVEM, GITRL, HLA-E, HLA-1, and markers that are involved in vascular protection, such as CD55 and CD39 (Figure 1). Levels of HLA-DR and CD59 were unaffected.

### 2.2. Results of Phenotypic Profiling in Human Primary Cell-Based BioMAP^®^ Systems

**Figure 2:**
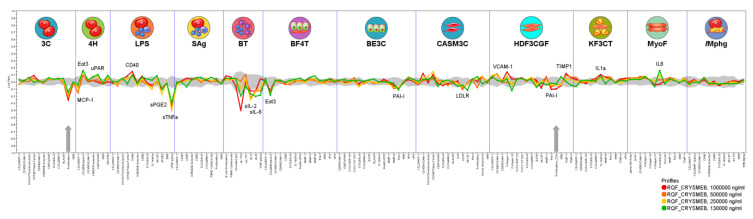
BioMAP^®^ profile of KLEPTOSE^®^ CRYSMEB in the Diversity PLUS^®^ panel. The x-axis lists the quantitative protein-based biomarker readouts measured in each system. The y-axis represents a log-transformed ratio of the biomarker readouts for the drug-treated sample (n = 1) over vehicle controls (n ≥ 6). The grey region around the y-axis represents the 95% significance envelope generated from historical vehicle controls. Antiproliferative effects are indicated by a thick grey arrow.

KLEPTOSE^®^ CRYSMEB profiling (Figure 2) at the indicated concentrations identified 16 modulated annotated readouts in the Diversity PLUS^®^ panel out of the 148 analyzed biomarker endpoints. No cytotoxicity was observed with KLEPTOSE^®^ CRYSMEB at any of the concentrations tested in this study. KLEPTOSE^®^ CRYSMEB was antiproliferative to human primary endothelial cells (at 1000 μg/mL, 500 μg/mL, 250 μg/mL, and 130 μg/mL) and fibroblasts (at 1000 μg/mL).

KLEPTOSE^®^ CRYSMEB impacted inflammation-related activities. This was seen as decreases in monocyte chemoattractant protein-1 (MCP-1), tumor necrosis factor-alpha (TNF-α), and prostaglandin E2 (PGE_2_) and increases in vascular cell adhesion molecule 1 (VCAM-1), interleukin 8 (IL-8), interleukin-1 alpha (IL-1α), modulation of eotaxin-3, immunomodulatory activities (decreases in interleukin 6 (IL-6), and interleukin 2 (IL-2), as well as increased CD40, tissue remodeling activities in the form of decreased plasminogen activator inhibitor-1 (PAI-1), increases in TIMP metallopeptidase inhibitor 1 (TIMP-1) and urokinase plasminogen activator receptor (uPAR), and decreased low-density lipoprotein receptor (LDLR), a cell surface receptor that is involved in cholesterol regulation that mediates endocytosis of low-density lipoprotein (LDL) levels (Figure 2).

**Figure 3:**
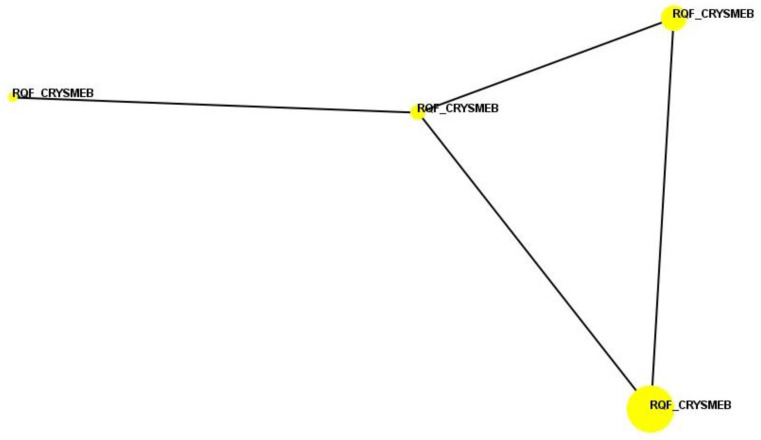
Cluster analysis (function similarity map) for KLEPTOSE^®^ CRYSMEB. Each colored circle represents the BioMAP^®^ profile at a specific concentration, with larger circles representing higher concentrations. Profiles that are similar with a Pearson’s correlation coefficient (r) ≥ 0.700 are connected by lines.

A similarity search analysis against the whole BioMAP^®^ reference database (Table 1) showed that the profile is most similar to that of VX-745 (when tested at 120 nM); the Pearson’s correlation coefficient between these two profiles is r = 0.540, which is below the predetermined threshold of r = 0.700, meaning that there is no statistically significant mechanistic similarity. VX-745 is an inhibitor of p38 MAPK, with greater selectivity for p38α versus p38β. The top similarity search match for the KLEPTOSE^®^ CRYSMEB profile at the second highest concentration (500 μg/mL) was quinacrine dihydrochloride dihydrate (when tested at 1.1 μM). The Pearson’s correlation coefficient between these two profiles is r = 0.562, which is also below the threshold for statistically significant mechanistic similarity. Quinacrine dihydrochloride dihydrate is an inhibitor of phospholipase A2 (PLA2) and is used as an antimalarial and anthelmintic agent. No similarity search matches were identified for KLEPTOSE^®^ CRYSMEB above the threshold for mechanistic similarity at any of the concentrations tested. In a pairwise correlation analysis, KLEPTOSE^®^ CRYSMEB clusters at all four concentrations with a Pearson’s correlation coefficient (r) ≥ 0.700, meaning the induced phenotype is maintained across the dilution range (Figure 3), a characteristic commonly observed in marketed drugs.

### 2.3. Results of the PBMC Model for Cytokine Secretion and Resolution Assays

#### 2.3.1. Cytokine Quantification

**Figure 4:**
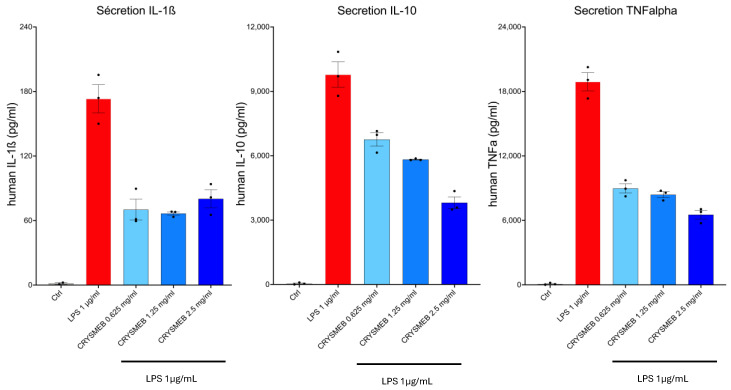
Cytokine quantification. IL-1β, IL-10, and TNF-α were quantified in cell culture supernatants: mean +/− SEM (n = 3) in pg/mL; control, LPS alone, or with KLEPTOSE^®^ CRYSMEB at three different doses.

KLEPTOSE^®^ CRYSMEB’s impact on cytokine production was assessed in a PBMC culture model stimulated by LPS (1 µg/mL). Figure 4 shows the quantification of IL-1β, IL-10, and TNF-α production (mean and SEM of three replicates). These results demonstrate that KLEPTOSE^®^ CRYSMEB inhibited the secretion of these three cytokines, with a dose-dependent effect on IL-10 and TNF-α.

#### 2.3.2. Lipid Mediator Quantification

KLEPTOSE^®^ CRYSMEB capacities to modulate inflammation and resolve lipid mediator production were investigated using PBMCs stimulated by PMA/A23187. The secretion of 23 lipid mediators was quantified (Table 2). We observed that KLEPTOSE^®^ CRYSMEB induced significant activation of pro-inflammatory mediators (5-HETE intermediate mediator, as well as a strong activation of PGE_2_ and TXB2). Of note was the fact that these activations were not seen to be dose-dependent. We also detected a significant increase in the production of pro-resolving 14-HDOHE and 17-HDOHE lipid intermediates, but no increase in SPM mediators by themselves. Interestingly, we also found that KLEPTOSE^®^ CRYSMEB induced an increase in 15-HETE and 12-HETE production, which suggested activation of the 12-LOX and 15-LOX pathways.

## 3. Discussion

Endothelial cells share common innate immune functions with macrophages, including cytokine secretion, antigen presentation, pathogen-associated molecular pattern (PAMP) sensing, and phagocytosis. Their plasticity allows them to regulate immune surveillance over the different tissues of the organism [35].

Interestingly, we report here that KLEPTOSE^®^ CRYSMEB can upregulate the expression of molecules that are involved in the crosstalk between immune cells and surrounding cells like endothelial cells. Indeed, the increase in HLA-1 expression elicited by this drug may be linked to immune cell recruitment, as HLA-1 signaling was reported to impact leukocyte tethering or neutrophil adherence [36]. Antigenic presentation through class I molecules (HLA-ABC and HLA-E) that govern innate and adaptative immune responses [21] improved.

This finding is supported by the drug-induced increase in ICAM-1, a protein that is implicated in the firm adhesion of activated T cells to the endothelium and their *trans*-endothelial migration [37], and GITRL, a molecule that is also involved in cell adhesion/migration/activation of innate cells [38,39,40]. Furthermore, the increase in GITRL on the surface of HUVECs (Figure 1E) may potentially facilitate GITR/GITRL signaling, thus resulting in T cell activation and proliferation [41,42]. GITR interaction stimulates antiviral responses through enhanced TNF-α and IFN-γ production in vivo [43] and GITR is expressed on numerous activated immune cell types such as T reg, macrophages, and natural killer (NK) cells, giving the opportunity for KLEPTOSE^®^ CRYSMEB to also interact with cells that are involved in mediating innate immunity [44]. To support this finding, we confirmed that HLA-E was constitutively expressed on endothelial cells, and it is well established that HLA-E–NK receptor interaction is crucial for NK cell activation [45,46]. Moreover, KLEPTOSE^®^ CRYSMEB induced an increase in IFN-γR1. IFN-γ binds to IFN-γR1 with a very high affinity to induce signaling via the JAK/STAT pathway and is constitutively expressed on endothelial cells. KLEPTOSE^®^ CRYSMEB induces overexpression of this receptor, which can potentially lead to stronger sensitization of cells to IFN-γ and thus better activation of these cells. IFN-γ is secreted by immune cells during the early phase of the immune response (NK, LT CD4 helper, and LT CD8), allowing dedicated antigen-presenting cells (e.g., dendritic cells and macrophages) to increase the presentation of peptides from pathogens on HLA class II molecules. Moreover, KLEPTOSE^®^ CRYSMEB increased both HLA-E and GITRL, a mechanism that can probably serve to help the immune system boost immunosurveillance upon infection [43]. Activation and proliferation of T cells can also be supported by HVEM expression in an HVEM/LIGHT receptor/ligand context [47]: HVEM plays a role in innate and adaptive immunity depending on the ligand: in the presence of LIGHT (LIGHT/HVEM), it induces a co-stimulation signal, while in the presence of BTLA (BTLA/HVEM), it acts as co-inhibitor of the response [47]. Since KLEPTOSE^®^ CRYSMEB increases HVEM under inflammatory conditions, it would be interesting to study the role of BTLA or LIGHT fixation in the presence of this cyclodextrin.

This first body of data supports the fact that KLEPTOSE^®^ CRYSMEB tends to stimulate the expression of endothelial cell markers triggering the recruitment, migration, co-stimulation, and activation of T cells and NK cells. However, it is also interesting to highlight vascular protection. Indeed, complement deposition at the cell surface level is a mechanism that is often used to protect the human body from pathogens by inducing lysis that is specific to the pathogen’s membrane with complement-dependent cytotoxicity [48]. Different pathways are involved in this system (classical, alternative, and lectin pathways) [49] and CD55 is ideally placed at the beginning of the process to inhibit complement cascade deposition. Indeed, the increase in CD55 in HUVECs when treated with KLEPTOSE^®^ CRYSMEB (Figure 1E) can protect endothelial cells from lysis by accelerating the degradation of C3 convertase, a central enzyme that regulates the complement cascade at the initial step, with CD59 interfering only at the terminal step (preventing the formation of Membrane Attack Complex C5b9) [50]. 

Moreover, the overexpression of CD55 inhibits complement activation; this could be of interest in Alzheimer’s disease (AD) since the deposition of complement is observed in the brains of patients with AD [51]. This observation is very interesting in the context of clinical uses in Phase 2 clinical trials of cyclodextrin derivatives for the treatment of AD [52]. 

Another way to protect endothelial cells from injury is to modulate platelet aggregation, a natural mechanism that occurs during bleeding. Interestingly, KLEPTOSE^®^ CRYSMEB demonstrated the capability to increase levels of CD39, an endopeptidase that prevents excessive platelet aggregation and vascular leakage [53,54,55]. Moreover, CD39 is also described as a new immunomodulatory protein and is compared with a new checkpoint inhibitor [56], a dual role that can very well illustrate KLEPTOSE^®^ CRYSMEB bioactivity in vascular cells.

Montecucco et al. [7] showed that KLEPTOSE^®^ CRYSMEB has an impact on atherosclerosis in mouse models of disease. In this disease, platelet accumulation induces the recruitment of inflammatory cells towards the lesion sites [56]. The impact of KLEPTOSE^®^ CRYSMEB on different markers and pathways that are involved in the modulation of platelet aggregation, preventing excessive platelet aggregation, cell lysis, and vascular leakage, could explain in part the observed in vivo impact of this cyclodextrin on atherosclerosis.

This first screening strategy using a primary endothelial cell (HUVEC) model demonstrated the in vitro potential of KLEPTOSE^®^ CRYSMEB to modulate the immune system and potentially manage vascular issues.

These impacts of KLEPTOSE^®^ CRYSMEB on cellular interactions between immune cells and vascular cells, as well as other cell types such as those from respiratory, muscle, and skin tissues, were elucidated using phenotypic profiling in human primary cell-based BioMAP^®^ systems. The observed decrease in prostaglandin E2 (PGE_2_) in the LPS system, which models monocyte activation, is shared by KLEPTOSE^®^ CRYSMEB and several NSAIDs when tested on this platform. This activity is a sentinel biomarker for COX inhibition and suggests that KLEPTOSE^®^ CRYSMEB has similar anti-inflammatory properties. Furthermore, KLEPTOSE^®^ CRYSMEB treatment led to decreases in tissue inflammation and immunomodulatory biomarkers such as IL-2, IL-6, MCP-1, and TNF-α. These effects on Th1 inflammation (LPS system) or the T-cell-dependent activation of B cells that occurs in germinal centers (BT system) can be compared with the systemic effect of KLEPTOSE^®^ CRYSMEB on Th1-mediated immunity that was observed in mice in an atherosclerotic context and in human T lymphocytes in vitro [7]. Indeed, KLEPTOSE^®^ CRYSMEB dose-dependently inhibited the proliferation of Th1 CD4+ cells, and methyl β-cyclodextrins can switch off the pro-inflammatory effects of IFN-γ on the vascular endothelium. The inhibition of Th1 immune responses has been described as a potential strategy for inhibiting chronic inflammation and atherogenesis in mice [57,58]. Nevertheless, in the BioMAP^®^ T cell activation model (T-cell-driven Th1 vascular inflammation model), no impact of KLEPTOSE^®^ CRYSMEB on T cell proliferation was observed. Moreover, according to Montecucco et al. [7], methyl β-cyclodextrins can modulate the immune response, switching off the pro-inflammatory effects of IFN-γ on the endothelium since IFN-γ can enhance the expression of adhesion molecules in endothelial cells that are needed for leukocyte–endothelial cell interaction. However, our results on human umbilical vein endothelial cells show that KLEPTOSE^®^ CRYSMEB can induce an increase in IFN-γ receptors. Consequently, the anti-inflammatory effects of KLEPTOSE^®^ CRYSMEB seem to be driven by pro-inflammatory cytokine secretion inhibition and not at the cellular level on different receptors such as IFN-γR1, or on the expression of molecules that are involved in the crosstalk of immune cells and endothelial cells such as HLA-1, ICAM-1, and GITRL.

Some of the observed activities of KLEPTOSE^®^ CRYSMEB are also related to pro-inflammatory activities in the acute phase, such as increased amounts of IL-8, a pro-inflammatory cytokine that is involved in the recruitment of neutrophils into acute inflammatory sites, as well as the increase in VCAM-1, a cell adhesion molecule that mediates the adhesion of monocytes and T cells into sites of inflammation. Eotaxin-3 was found to be context-dependently modulated: it was increased in a Th2-type vascular inflammation model (4H) but decreased in a Th2-type lung inflammation model (BF4T). Whilst the stimulation environments of these two systems are relatively similar, the cell types are different, suggesting that KLEPTOSE^®^ CRYSMEB can have different impacts on different tissue types. Eotaxin-3 is a chemokine that mediates the recruitment of eosinophils and basophils into sites of tissue inflammation. This finding confirms the results of human umbilical vein endothelial cells regarding the impact of KLEPTOSE^®^ CRYSMEB on the modulation of the expression of molecules that are involved in the crosstalk between immune and endothelial cells.

This effect of KLEPTOSE^®^ CRYSMEB on immune responses seems to be at two distinct levels: affecting cytokine secretion and on the levels of cellular proteins that are involved with immune cell communication, recognition, etc.

A similarity search analysis against the whole BioMAP^®^ reference database, with thousands of molecules and reference agents of a wide array of mechanistic types previously profiled, revealed no significantly similar profile matches. This suggests that the mechanism of action of KLEPTOSE^®^ CRYSMEB is not shared with any molecule that has been tested as part of the reference database. Moreover, no other beta-cyclodextrins or their derivatives were tested at concentrations deemed to be active, and thus profiles relating to this are not present within the BioMAP^®^ reference database. As such, this could potentially represent a unique mechanism of action, although the exact nature of this would need to be confirmed in a follow-up study of a more detailed mechanistic nature.

**Figure 5:**
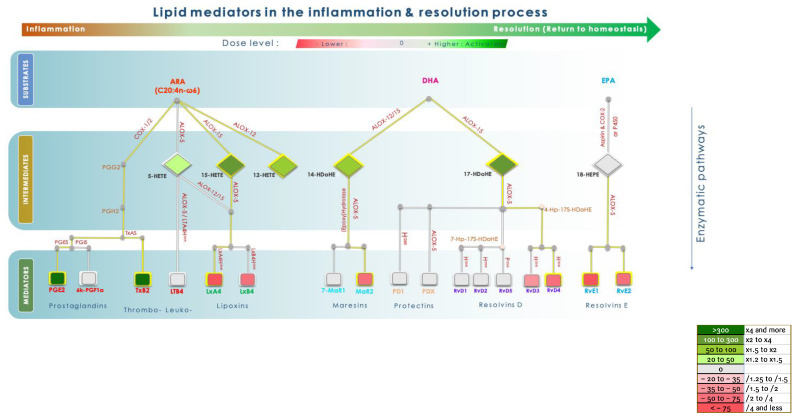
Summary of metabolic pathway modulation for ARA, DHA, and EPA on PBMCs when exposed to KLEPTOSE^®^ CRYSMEB.

In addition, KLEPTOSE^®^ CRYSMEB inhibited the secretion of IL-1β, IL-10, and TNF-α in PBMCs when stimulated by LPS (Figure 4). This suggests that this compound has anti-inflammatory capabilities, especially via the reduction of cytokines involved in inflammatory modulation: IL-1β and TNF-α as pro-inflammatory cytokines, and IL-10 with its pivotal role in immune modulation (IL-10 acts at the same time as an anti-inflammatory cytokine by protecting the body from an uncontrolled immune response and as an immunostimulating mediator [59]). Moreover, when testing under the same condition (Supplementary Materials Figure S1), the anti-inflammatory compound (Paracetamol, NSAID Ibuprofen 100 µM and Corticosteroid Dexamethasone 1 µM), IL-1β, IL-10, and TNF-α were markedly decreased. At first glance, these results would suggest that although the effects occurred at the highest doses, this methylated cyclodextrin acted as an anti-inflammatory compound. To compare with NSAID’s mechanism of action, KLEPTOSE^®^ CRYSMEB’s impact was then investigated by studying the inflammatory and resolving lipid mediator metabolism pathways activated in PBMCs when stimulated by PMA/A23187. Surprisingly, KLEPTOSE^®^ CRYSMEB activated the COX enzyme as well as the 5-LOX, 12-LOX, and 15-LOX pathways and induced an increase in pro-inflammatory mediators (metabolic pathway modulations corresponding to these results are represented in Figure 5). These results are in accordance with Choi et al., 2004 [60], who showed that methyl-beta-cyclodextrin induced COX-2 expression, PGE_2_ synthesis, and COX-2 promoter activity in a dose- and time-dependent manner. However, our results showed an increase in pro-inflammatory mediators at a dose of 1.25 mg/mL, but the values obtained with the highest tested dose (2.5 mg/mL) were lower. We hypothesize that KLEPTOSE^®^ CRYSMEB could complex with inflammatory mediators at high doses. Indeed, other authors showed a reduction in inflammation due to the complexation of RAMEB with PGE_2_ [61]. Our results for PBMCs alone are the opposite of those found with the BioMAP^®^ LPS system, in which a diminution of PGE_2_ levels was observed. However, various experimental conditions in those studies were different and could explain this apparent contradiction: First, the stimulant used was PMA/A23187 (versus LPS), which induces a much stronger inflammatory response. Then, the lipid mediators were measured 1 h after PMA/A23187 stimulation (versus 48 h and 24 h for the assays in HUVECs and 24, 48, 72, and 168 h in the BioMAP^®^ models). Finally, the PBMCs were not in contact with HUVECs, as was the case in the BioMAP^®^ system. KLEPTOSE^®^ CRYSMEB could indeed demonstrate better efficacy at a later point and on milder inflammation (as is the case with LPS stimulation). Moreover, the difference in excreted PGE_2_ could be linked to the method of stimulation of inflammation in the two assays since KLEPTOSE^®^ CRYSMEB can bind to the lipid part of LPS, hindering TLR signaling.

Additionally, the presence of vascular endothelial cells could influence the response to KLEPTOSE^®^ CRYSMEB exposure and the impact on the crosstalk between immune cells and other cell types. Moreover, we did not observe the production of specialized pro-resolving lipid mediators (SPMs) despite the production of pro-resolving lipid intermediates such as 14-HDoHe and 17-HDoHe. One reason that could explain these observations is that inflammation is a kinetic process, characterized by the sequential production of pro-inflammatory and pro-resolving mediators. We thus suggest that the quantification of SPMs at other time points would allow for better deciphering of the mechanisms of action of KLEPTOSE^®^ CRYSMEB. Moreover, the impact of this cyclodextrin on lipid mediators could influence its response to the inflammation process. It would be interesting to assess the inhibitory effect of KLEPTOSE^®^ CRYSMEB.

Finally, it seems that the anti-inflammatory impact of KLEPTOSE^®^ CRYSMEB depends on the way in which the stimulation of this inflammation is generated, especially when using LPS. Indeed, according to Chen et al., 2021 [62], methyl-β-cyclodextrin suppressed the monocyte-endothelial adhesion on HUVECs induced by LPS/oxidized LDL (oxLDL) by inhibiting the expression of adhesion molecules (ICAM-1 and VCAM-1) via signaling pathways involving LPS, IκB kinase, NF-κB, or oxLDL-Akt-NF-κB.

This effect on the adhesion molecules ICAM-1 and VCAM-1 was not in line with our results on HUVEC and BioMAP^®^ models since we saw an increase in these adhesion factors. Additionally, according to Kitagawa et al., 2002 [63], a decrease in ICAM-1 can limit the progression of atherosclerosis. Thus, the anti-inflammatory effect of KLEPTOSE^®^ CRYSMEB observed in vivo during the atherosclerosis process [7] could be counterbalanced by this effect on ICAM-1. Conversely, according to Wang et al., 2019 [18], the expression of ICAM-1 in human patients suffering from atherosclerosis could be positively linked with the stability of coronary atherosclerotic plaques. Furthermore, it is interesting to note that the stimulation of ICAM-1 in association with OX40L can contribute to atherosclerotic plaque stability [18]. 

Another possible mode of action of KLEPTOSE^®^ CRYSMEB may be an effect on membrane cells’ cholesterol depletion leading to a decrease in cytokine expression. Indeed, Horlock et al., (2022) [64] showed that methyl-β-cyclodextrin depletes cholesterol and induces a decrease in LPS-stimulated secretion of IL-1α, IL-1β, and IL-8 from bovine granulosa cells. Finally, we must consider another possible impact of KLEPTOSE^®^ CRYSMEB on the inflammatory process, especially the possible binding of this cyclodextrin to the lipid part of LPS, hindering TLR signaling.

In addition, KLEPTOSE^®^ CRYSMEB induced a decrease in low-density lipoprotein receptors, which are involved in cholesterol regulation. This is due to the well-known membrane-cholesterol-extracting effect of cyclodextrins and their derivatives on cholesterol levels and thus their impact on lipid raft disruption, as well as changes in biophysical parameters and organization of membrane bilayers, which can also induce an indirect modulation of protein function [11]. In summary, this also represents another facet of KLEPTOSE^®^ CRYSMEB activity that would warrant further evaluation and potential confirmation in follow-up studies.

**Figure 6:**
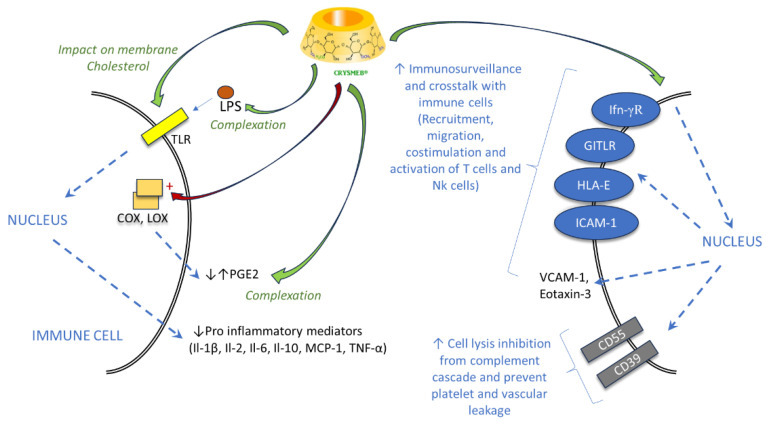
Summary of the mechanism of KLEPTOSE^®^ CRYSMEB’s effect on inflammatory responses.

Regarding the results obtained using different cellular models (Figure 6), KLEPTOSE^®^ CRYSMEB induced an anti-inflammatory impact, which could be due to different mechanisms: (i) a direct effect on TLR or indirect effect due to complexation of cholesterol in lipid rafts at the membrane level, (ii) a complexation of the lipid part of LPS, which leads to a decrease in TLR response, and (iii) complexation of pro-inflammatory mediators such as prostaglandins (PGE2). All these phenomena lead to a decrease in inflammatory mediators, which proves an anti-inflammatory effect. Conversely, this methylated cyclodextrin could stimulate a pro-inflammatory response involving the synthesis of enzymes such as cyclooxygenase (COX) and PGE2 and on proteins involved in communications with immune cells such as ICAM-1, GITLR, etc., probably due to the stress mechanism via interaction with membrane cholesterol. Moreover, KLEPTOSE^®^ CRYSMEB induced overexpression of CD55 involved in complement cascade inhibition and CD39, which is described as a new immunomodulatory checkpoint inhibitor protein.

Finally, KLEPTOSE^®^ CRYSMEB, like other cyclodextrins, acts as a scavenger of pro-inflammatory compounds such as LPS or inflammatory mediators but not as a pharmacologically active molecule such as corticosteroids or NSAIDs. In this way, it could act as an anti-inflammatory compound. Nevertheless, the in vivo impact for anti-inflammatory purposes may show some limits. Indeed, although this product is well tolerated in vivo (no observed adverse effect level in rats 28 days after daily intravenous administration of 600 mg/Kg/day [65]), the doses to be used to obtain effects comparable to NSAIDs or corticosteroids would be much higher. In addition, the poor oral bioavailability of KLEPTOSE^®^ CRYSMEB would limit the use of this product via the parenteral route (0.69% in rats; [65]). Moreover, the in vivo dosage of KLEPTOSE^®^ CRYSMEB must be evaluated considering its impact on the inflammation process, especially the stimulation of pro-inflammatory responses (involving lipid mediators and communication proteins between immune cells). KLEPTOSE^®^ CRYSMEB could be used as a therapeutic agent based on its ability to actively extract lipids from cell membranes, to provide a suitable carrier system for drug delivery, and for its anti-inflammatory impact, especially for diseases such as cardiovascular diseases (atherosclerosis), lipid disorders (NASH, NAFLD), and neurodegenerative diseases (Alzheimer’s disease, Parkinson’s disease), which involve important inflammatory processes [6,7,8].

## 4. Methods and Materials

All tested samples were of pharmaceutically active substance quality. Methylated cyclodextrin Crysmeb (KLEPTOSE^®^ CRYSMEB) and Isosorbide (ISOSORBIDE C PHARMA) were supplied by Roquette Frères (Lestrem, France).

### 4.1. HUVEC Immuno-Profiling

Primary human umbilical vein endothelial cells (HUVECs, PromoCell GmbH, Heidelberg, Germany) (passage 4) were seeded at 5000 cells per well in 96-well plates in ECGM medium (PromoCell GmbH, Heidelberg, Germany) supplemented with 2% FBS and grown for 4 days before adding KLEPTOSE^®^ CRYSMEB or ISOSORBIDE C PHARMA (a polyol used as a humectant and in medicine as an osmotic diuretic without known pharmacological effects) to the medium at non-cytotoxic concentrations of 2.5 mg/mL ). The cells were treated for 48 h in the presence (“inflammatory condition”) or absence (“non-inflammatory condition”) of 10 ng/mL TNF-α or 20 ng/mL IFN-γ before multi-parametric immunostaining and flow cytometry analysis. Briefly, detached cells were immunolabelled in FACS buffer (PBS/BSA 1%/EDTA 2 mM) with the following antibodies directed against multiple targets for 30 min on ice: ICAM-1, GITRL, HLA-E, CD55, CD59, CD39, VCAM-1, HVEM, IFN-γR1, TNFR1, HLA-ABC, and HLA-DR (all purchased from BioLegend, San Diego, CA, USA). Cells were analyzed by flow cytometry on day 2 on a BD FACS Canto II, configuration 4/2/2. One experiment was performed in triplicate for each condition.

### 4.2. Phenotypic Profiling in Human Primary Cell-Based BioMAP^®^ Systems

Phenotypic profiling with the BioMAP^®^ Diversity PLUS^®^ panel was conducted by Eurofins Discovery (St Charles, MO, USA) as described previously [28], with doses of KLEPTOSE^®^ CRYSMEB of 1000 µg/mL, 500 µg/mL, 250 µg/mL, and 130 µg/mL.

Details of the biology of each system within the BioMAP^®^ Diversity PLUS^®^ panel have been published previously and are available on the Eurofins Discovery website (https://www.eurofinsdiscovery.com/solution/biomap-diversity-plus, accessed on 21 May 2024) [30]. The system details are also given in Table 3.

#### Human Primary Cells

All studies follow the guidelines for human subject research under United States HHS human subject regulations (45 CFR Part 46). The preparation and culture of primary human cell types and methods used for the systems included were as previously described [29]. All primary human cells used in this work were obtained from commercial sources, cultured according to the supplier’s recommendation, and used at early passage (≤P4) to minimize adaptation to cell culture conditions and to preserve physiological signaling responses. Furthermore, cells were pooled from multiple (three or more) donors in this study to minimize the effect of donor-to-donor variation.

Primary human cell types used in BioMAP^®^ systems and their stimuli include the following: 3C System (HUVEC/IL-1β, TNF-α, and IFN-γ), 4H System (HUVEC/IL-4 and histamine), LPS System (PBMC and HUVEC/LPS), SAg System (PBMC and HUVEC/TCR ligands), BT System (CD19+ B cells and PBMC/anti-IgM and TCR ligands), BE3C System (bronchial epithelial cells/IL-1β, TNF-α, and IFN-γ), BF4T System (bronchial epithelial cells and human dermal fibroblasts/TNF-α and IL-4), HDF3CGF System (human dermal fibroblasts/IL-1β, TNF-α, IFN-γ, EGF, basic-FGF, and PDGF-BB), KF3CT System (keratinocytes and dermal fibroblasts/IL-1β, TNF-α, and IFN-γ), CASM3C System (coronary artery smooth muscle cells/IL-1β, TNF-α, and IFN-γ), MyoF System (differentiated lung myofibroblasts/TNF-α and TGF-β), and Mphg System (HUVECs and macrophages/TLR2 ligands). The concentrations of stimuli were as previously published [28]. Adherent cells were cultured to confluence prior to assay initiation. The numbers of lymphocytes used were as previously published. Assays were initiated by the addition of KLEPTOSE^®^ CRYSMEB, followed by the addition of appropriate stimuli. Assay plates were then incubated at 37 °C in 5% CO_2_ for 24 h, or as otherwise indicated. KLEPTOSE^®^ CRYSMEB solution, prepared in DMSO (final concentration ≤ 0.1%), was added at the previously indicated concentrations 1 h before stimulation and remained in culture for 24 h or as otherwise indicated (48 h MyoF system; 72 h BT system soluble readouts; 168 h BT system secreted IgG). Cell proliferation was determined by sulforhodamine B (SRB) assay for adherent cell types or AlamarBlue™ for PBMCs, B cells, and T cells [29]. For proliferation assays, individual cell types were cultured at sub-confluence and measured at specific times for different primary cell types (48, 72, or 96 h). After stimulation, plates and supernatants were harvested and biomarkers were measured by ELISA and other methodologies. The levels of biomarker endpoints were measured by ELISA as described [29,66,67]. The SRB assay was performed by staining cells with 0.1% sulforhodamine B after fixation with 10% TCA and reading of wells at 560 nm [29]. PBMC viability was assessed by adding AlamarBlue™ to PBMCs that had been cultured for 24 h in the presence of activators and compounds, and subsequent measurement of its reduction after 8 h.

Drug controls were added to each plate (colchicine 1.1 μM), non-stimulated wells, and vehicle controls (0.1% DMSO). All data are normalized to the stimulated in-plate vehicle control wells.

Raw data values for each readout for KLEPTOSE^®^ CRYSMEB were divided by the raw mean vehicle control and then log10-transformed as previously described [68]. Significance envelopes at a 95% level, calculated from historical controls, were used to classify activities as “annotated” when two or more consecutive concentrations changed in the same direction relative to the vehicle controls, were outside of the significance envelope, and had at least one concentration with an effect size >20% (|log10 ratio| > 0.1). All four profiles were compared for shape similarity against the BioMAP^®^ reference database (>4500 test agents overall; no other beta-cyclodextrins or derivatives were tested at concentrations deemed to be active and thus profiles relating to this are not present within the BioMAP^®^ reference database) as previously described [29,68]. Cluster analysis was performed for the four test agent concentration profiles to find the “proximity” of each profile from one another. Profiles that are similar with a Pearson’s correlation coefficient (r) ≥ 0.7 are considered to have mechanistically relevant similarity and are connected by lines in Figure 3.

### 4.3. Peripheral Blood Mononuclear Cell Model for Cytokine Secretion and Resolution Assays

#### 4.3.1. Peripheral Blood Mononuclear Cells

Peripheral blood mononuclear cells (PBMCs) from healthy volunteers were obtained from l’Etablissement Français du Sang (EFS, Toulouse, France). After isolation, the PBMCs were frozen until used in the experiments. Cell culture medium Macrophage-SFM (M-SFM) was purchased from Gibco (Illkirch, France). LPS from the *Escherichia coli* K12 strain was bought from Invivogen (Toulouse, France). Antibiotics, phorbol 12-myristate 13-acetate (PMA), and A23187 (calimycin) were bought from Millipore Sigma (Saint-Quentin Fallavier, France). Multiplexed kits for cytokine quantification (IL-1β, IL-10, and TNF-α) were bought from Millipore Sigma. 

#### 4.3.2. Cell Isolation and Culture

PBMCs were obtained from healthy blood donor buffy coats by a standard Ficoll–Hypaque gradient method. PBMCs were seeded in Macrophage-SFM with antibiotics at 37 °C in a humidified atmosphere containing 5% CO_2_. PBMCs were exposed to test compounds and inhibitors for 24 h. Medium was then changed to perform the resolution assay.

#### 4.3.3. Lipidomic Analysis for Resolution Assay

HBSS +/+ buffer was used for the resolution assay, with renewal of the test compounds and inhibitors. Cells were exposed to the inflammatory stimulus (PMA/A23187) for 1 h. Culture supernatants were then collected for subsequent analysis of lipid mediators. The extraction protocol and LC-MS/MS analysis were performed as adapted from Le Faouder et al. [33]. Briefly, samples were extracted using oasis HLB 96-well solid phase extraction (Waters, Saint Quentin en Yvelines, France). LC-MS/MS analysis was performed on a UHPLC system (EXION LC AD, SCIEX, Villebon sur Yvette, France) coupled to a QTrap 6500+ MS (SCIEX) instrument equipped with electrospray ionization operating in negative mode.

#### 4.3.4. Cytokine Secretion Assay

PBMCs were incubated with KLEPTOSE^®^ CRYSMEB for 24 h in the presence of antibiotics. The culture medium with KLEPTOSE^®^ CRYSMEB was then renewed, and the PBMCs were stimulated with LPS 1 µg/mL for 24 h. The supernatants were then collected and frozen until analysis. Cytokines (IL-1β, IL-10, and TNF-α) were analyzed with a MILLIPLEX^®^ Multiplex assay kit and measured on a Luminex MAGPIX^®^ reader (Luminex corporation, Austin, TX, USA).

#### 4.3.5. Statistical Evaluation

The results were expressed as mean ± SEM; the mean of the groups was compared using One-way ANOVA followed by Tukey’s multiple comparisons test. In no group comparisons, an unpaired two-tailed *t*-test was used. All Statistical analyses were performed using GraphPad Prism^®^ version 10.2.3 for Windows (GraphPad Software^®^, Boston, MA, USA).

## 5. Conclusions

KLEPTOSE^®^ CRYSMEB can modulate the immune system in vitro and potentially manage vascular issues by stimulating the expression of molecules that are involved in the crosstalk between immune cells and other cell types, especially for the recruitment, migration, co-stimulation, and activation of T cells and NK cells. KLEPTOSE^®^ CRYSMEB showed anti-inflammatory effects driven by the inhibition of pro-inflammatory cytokine secretion and not at the cellular level on different receptors. As a result, the effect of KLEPTOSE^®^ CRYSMEB on immune responses seems to be at two distinct levels: cytokine secretion and proteins that are involved in communications with immune cells; thus, this compound could have different impacts on different tissue types. 

Finally, regarding the results obtained using different cellular models, KLEPTOSE^®^ CRYSMEB showed an anti-inflammatory impact, which could be due to its effect on receptors such as TLR or direct complexation of LPS or PGE_2_, and conversely, this methylated cyclodextrin could stimulate a pro-inflammatory response involving lipid mediators and proteins involved in communications with immune cells probably via interaction with membrane cholesterol.

Moreover, the mechanism of action of KLEPTOSE^®^ CRYSMEB seems to not be shared with well-known anti-inflammatory molecules such as NSAIDs or corticosteroids. Additionally, it seems to have an impact on signaling pathways involving LPS via an effect on membrane cells’ cholesterol depletion and possibly via interacting directly with LPS or lipid mediators. Further studies are needed to elucidate the detailed mechanistic nature of this, especially the interaction between immune cells and other cell types and the complexation mechanism with LPS or lipid mediators such as PGE_2_. Thus, it could be interesting to investigate the effects of KLEPTOSE^®^ CRYSMEB on the inflammation mechanism using non-lipidic inflammatory agents to elucidate the impact on the anti-inflammatory mechanism versus lipid mediators and the LPS-entrapping phenomenon. Moreover, it would be important to study the kinetic aspects and the preventive and/or curative effects in order to better dissect and characterize the mechanism(s) of action of KLEPTOSE^®^ CRYSMEB. Additionally, other investigations are also needed to determine if this balance between the pro- and anti-inflammatory response is dose-dependent. Finally, the stimulation of CD55 expression induced by this methylated cyclodextrin should be assessed in the AD context.

## Figures and Tables

**Table 1:** KLEPTOSE^®^ CRYSMEB similarity search analysis against the whole BioMAP^®^ reference database of >4500 test agents.

KLEPTOSE^®^ CRYSMEB Concentration (µg/L)	Database Match	Biomap Z-Standard	Pearson’s Score	Number of Common Readouts	Mechanism Class
1000	VX745, 120 mM	7.279	0.540	148	P38 MAPK inhibitor
	VX745, 370 mM	7.122	0.531	148	P38 MAPK inhibitor
	SB203580, 3.3 µM	7.109	0.530	148	P38 MAPK inhibitor
500	Quinacrine dihydrochloride dihydrate, 1.1 µM	7.655	0.562	148	Phospholipase A2 inhibitor
	Tyloxapol, 5 µM	7.503	0.656	148	Surfactant agent
	Sodium hydroxide, 150 µM	7.283	0.540	148	Inorganic compound
250	Sodium hydroxide, 150 µM	7.852	0.573	148	Inorganic compound
	Pirfenidone, 190 µM	7.729	0.566	148	Antifibrotic agent
	Pirfenidone, 330 µM	7.541	0.555	148	Antifibrotic agent
130	Sildenafil citrate, 30 µM	7.259	0.539	148	PDE inhibitor
	Bemcentinib, 190 nM	7.157	0.533	148	AXL inhibitor
	Bemcentinib, 750 nM	7.131	0.531	148	AXL inhibitor

**Table 2:** Quantification of pro-inflammatory and pro-resolving lipid mediators. The table shows the mean values (n = 3) for each lipid mediator under each studied condition in pg/mL: control, PMA/A23187 alone or with KLEPTOSE^®^ CRYSMEB at 3 different doses. The presence of “-” indicates that the value was under the limit of quantification (the limit of quantification is indicated at the bottom of each column). * data significance (*p* < 0.05 compared to the control). Values in parentheses indicate the % of activation compared with PMA/A23187 stimulation. These values were calculated based on the mean values. (A) Metabolites originating from arachidonic acid (ARA) metabolism. (B) Metabolites originating from docosahexaenoic acid (DHA) metabolism. (C) Metabolites originating from eicosapentaenoic acid (EPA) metabolism.

A; ARA Metabolites (% Activation of PMA/A23187)													
Mean of n = 3 (pg/mL)				Inflammatory Mediators:								SPM:	
Doses (mg/mL)			Stimulation	PGE_2_	6K-PGF1A	TXB2	LTB4		5-HETE	12-HETE	15-HETE	LXA4	LXB4
Control	0		No	1.17 *	-	7.99 *	4.61		6.73 *	135.47 *	10.94 *	1.14 *	0.0125 *
				(−93.3)	-	(−98.4)	(−100.0)		(−99.9)	(−99.5)	(−96.9)	(−86.6)	(−99.9)
Control	0		Yes	17.56	-	502.97	12,488.17		10,061.78	28,236.36	355.95	8.50	10.42
				(0.0)	-	(0.0)	(0.0)		(0.0)	(0.0)	(0.0)	(0.0)	(0.0)
CRYSMEB	0.625		Yes	50.97 *	-	2465.47 *	13,852.19		12,709.66 *	42,393.25 *	790.23 *	6.57 *	11.51
				(190.2)	-	(390.2)	(10.9)		(26.3)	(50.1)	(122.0)	(−22.7)	(10.5)
CRYSMEB	1.25		Yes	222.70	-	4735.65	14,612.74		17,096.85 *	49,679.45 *	1036.83 *	7.10	6.55
				(1168.2)	-	(841.5)	(17.0)		(69.9)	(75.9)	(191.3)	(−16.5)	(−37.1)
CRYSMEB	2.5		Yes	113.47 *	-	3357.35 *	10,047.64		13,621.02	46,766.97 *	972.6 *	4.13 *	5.46
				(546.1)	-	(567.5)	(−19.5)		(35.4)	(65.6)	(173.2)	(−51.5)	(−47.6)
			LOQ (pg/mL)	0.313	5.000	0.625	0.625		0.625	1.250	1.250	0.156	1.250
**B** **; DHA Metabolites (% Activation of PMA/A23187 Control)**													
**Mean of n = 3 (pg/mL)**			**Intermediate** **Mediators:**		**SPM:**								
**Doses (mg/mL)**	**Stimulation**		**14-HDOHE**	**17-HDOHE**	**7(R)-MAR1**	**MAR2**	**PDX**	**PD1**	**RVD1**	**RVD2**	**RVD3**	**RVD4**	**RVD5**
Control	0	No	38.65 *	11.41 *	-	0.06 *	-	-	-	-	0.91	0.85	0.23 *
			(−96.3)	(−85.2)	-	(−96.9)	-	-	-	-	(−19.2)	(−16.1)	(−95.9)
Control	0	Yes	1054.67	77.28	-	1.84	-	-	-	-	1.13	1.01	5.63
			(0.0)	(0.0)	-	(0.0)	-	-	-	-	(0.0)	(0.0)	(0.0)
CRYSMEB	0.625	Yes	1848.19 *	148.6 *	0.92 *	1.27 *	-	-	-	-	1.15	0.98	6.59
			(75.2)	(92.3)	-	(-30.8)	-	-	-	-	(2.0)	(−2.9)	(17.0)
CRYSMEB	1.25	Yes	2070.57 *	152.1 *	0.97 *	1.1 *	-	-	-	-	0.6 *	0.81	7.13
			(96.3)	(96.8)	-	(−40.2)	-	-	-	-	(−46.8)	(−19.8)	(26.6)
CRYSMEB	2.5	Yes	1997.09 *	165.85 *	0.69	1.05 *	-	-	-	-	0.84	0.53 *	5.20
			(89.4)	(114.6)	-	(−42.7)	-	-	-	-	(−25.6)	(−47.4)	(−7.6)
		LOQ (pg/mL)	1.250	2.500	0.313	0.156	0.313	0.313	0.625	0.625	0.156	0.156	0.625
**C** **; EPA Metabolites (% Activation of PMA/A23187)**													
**Mean of n = 3 (pg/mL)**						**Intermediate** **Mediators:**				**SPM:**			
**Doses (mg/mL)**				**Stimulation**		**18-HEPE**				**RVE1**		**RVE2**	
Control		0		No		6.75 *				3.95		7.50	
						(−44.7)				(17.2)		(−8.8)	
Control		0		Yes		12.21				3.37		8.23	
						(0.0)				(0.0)		(0.0)	
CRYSMEB		0.625		Yes		11.20				1.45 *		7.28	
						(−8.3)				(−56.8)		(−11.5)	
CRYSMEB		1.25		Yes		10.86				1.73 *		5.53 *	
						(−11.1)				(−48.7)		(−32.8)	
CRYSMEB		2.5		Yes		11.26				1.13 *		4.82 *	
						(−7.8)				(−66.5)		(−41.4)	
				LOQ (pg/mL)		0.313				0.625		0.625	

**Table 3:** BioMAP^®^ systems tested in this study.

System	Icon	Cell Type	Stimulation	Disease Relevance	Biomarkers Readouts
3C	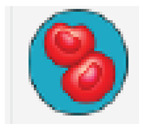	Venular endothelial cells	Il1-β, TNF-α, and INFγ	Cardiovascular Disease, Chronic Inflammation	MCP-1, VCAM-1, TM, TF, ICAM-1, E-selectin, uPAR, IL-8, MIG, HLADR, Proliferation, SR
4H	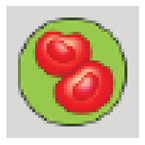	Venular endothelial cells	Il-4 and Histamine	Asthma, Allergy, Autoimmune Disease, Atopic Disease	MCP-1, Eotaxin-3, VCAM-1, P-selectin, uPAR, SRB, VEGFR2
LPS	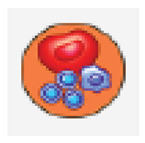	PBMC/Venular endothelial cells	TLR4 ligand	Cardiovascular Disease, Chronic Inflammation	MCP-1, VCAM-1, TM, TF, CD40, E-selectin, CD69, IL-8, IL-1α, M-CSF, sPGE2, SRB, sTNFα
SAg	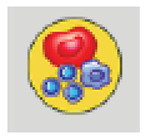	PBMC/Venular endothelial cells	TCR ligands (1×)	Autoimmune Disease, Chronic Inflammation	MCP-1, CD38, CD40, E-selectin, CD69, IL-8, MIG, PBMC Cytotoxicity, Proliferation, SRB
BT	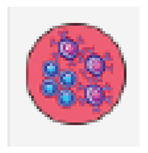	B cells/PBMC	A-IgM, TCR ligands (0.001×, sub-mitogenic levels)	Autoimmune Disease, Inflammation	B cell Proliferation, PBMC Cytotoxicity, Secreted IgG, sIL-17A, sIL-17F, sIL-2, sIL-6, sTNF α
BF4T	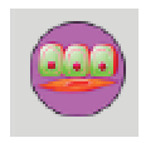	Bronchial epithelial cells/Dermal fibroblasts	Il4, TNF-α	Asthma, Allergy, Lung Inflammation, Atopic Disease	MCP-1, Eotaxin-3, VCAM-1, ICAM-1, CD90, IL-8, IL-1α, Keratin 8/18, MMP-1, MMP-3, MMP-9, PAI-1, SRB, tPA, uPA
BE3C	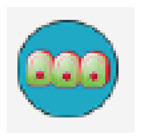	Bronchial epithelial cells	Il1-β, TNF-α, and INFγ	Lung Inflammation, COPD	ICAM-1, uPAR, IP-10, I-TAC, IL-8, MIG, EGFR, HLA-DR, IL-1α, Keratin 8/18, MMP-1, MMP-9, PAI-1, SRB, tPA, uPA
CASM3C	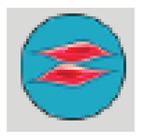	Coronary artery smooth muscle cells	Il1-β, TNF-α, and INFγ	Cardiovascular Inflammation, Restenosis	MCP-1, VCAM-1, TM, TF, uPAR, IL-8, MIG, HLA-DR, IL-6, LDLR, M-CSF, PAI-1, Proliferation, SAA, SRB
HDF3CGF	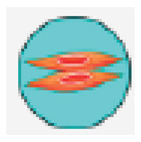	Dermal fibroblasts	Il1-β, TNF-α and INFγ, EGF, bFGF, PDGF-BB	Fibrosis, Chronic Inflammation, Wound Healing, Tissue Remodeling, Matrix Modulation	MCP-1, VCAM-1, ICAM-1, Collagen I, Collagen III, IP-10, I-TAC, IL-8, MIG, EGFR, M-CSF, MMP-1, PAI-1, Proliferation, SRB, TIMP-1, TIMP-2
KF3CT	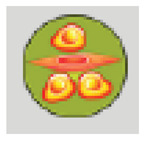	Keratinocytes/Dermal fibroblasts	Il1-β, TNF-α, and INFγ, TGF-β	Psoriasis, Dermatitis, Skin Biology	MCP-1, ICAM-1, IP-10, IL-8, MIG, IL-1α, MMP-9, PAI-1, SRB, TIMP-2, uPA
MyoF	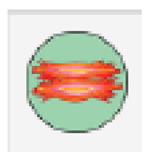	Lung fibroblasts	TNF-α and TGF-β	Fibrosis, Chronic Inflammation, Matrix Remodeling	a -SM Actin, bFGF, VCAM-1, Collagen I, Collagen III, Collagen IV, IL-8, Decorin, MMP-1, PAI-1, TIMP-1, SRB
/Mphg	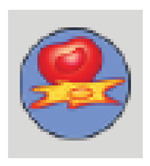	Venular endothelial cells/Macrophages	TLR2 ligand	Cardiovascular Inflammation, Restenosis, Chronic Inflammation	MCP-1, MIP-1α, VCAM-1, CD40, E-selectin, CD69, IL-8, IL-1α, M-CSF, sIL-10, SRB, SRB-Mphg

## Data Availability

The data presented in this study are available on request from the corresponding author due to legal reasons.

## Supplementary Materials

The following supporting information can be downloaded at: https://www.mdpi.com/article/10.3390/ijms25179748/s1.
