# Supplementary material for: Impact of Methylated Cyclodextrin KLEPTOSE® CRYSMEB on Inflammatory Responses in Human In Vitro Models
Source: Int J Mol Sci. 2024 Sep 9;25(17):9748. doi: 10.3390/ijms25179748 (PMC11396153; doi:10.3390/ijms25179748)
Supplement: Supplementary file 1 [file ijms-25-09748-s001.zip › ijms-3139481-supplementary.pptx]

## Slide 1
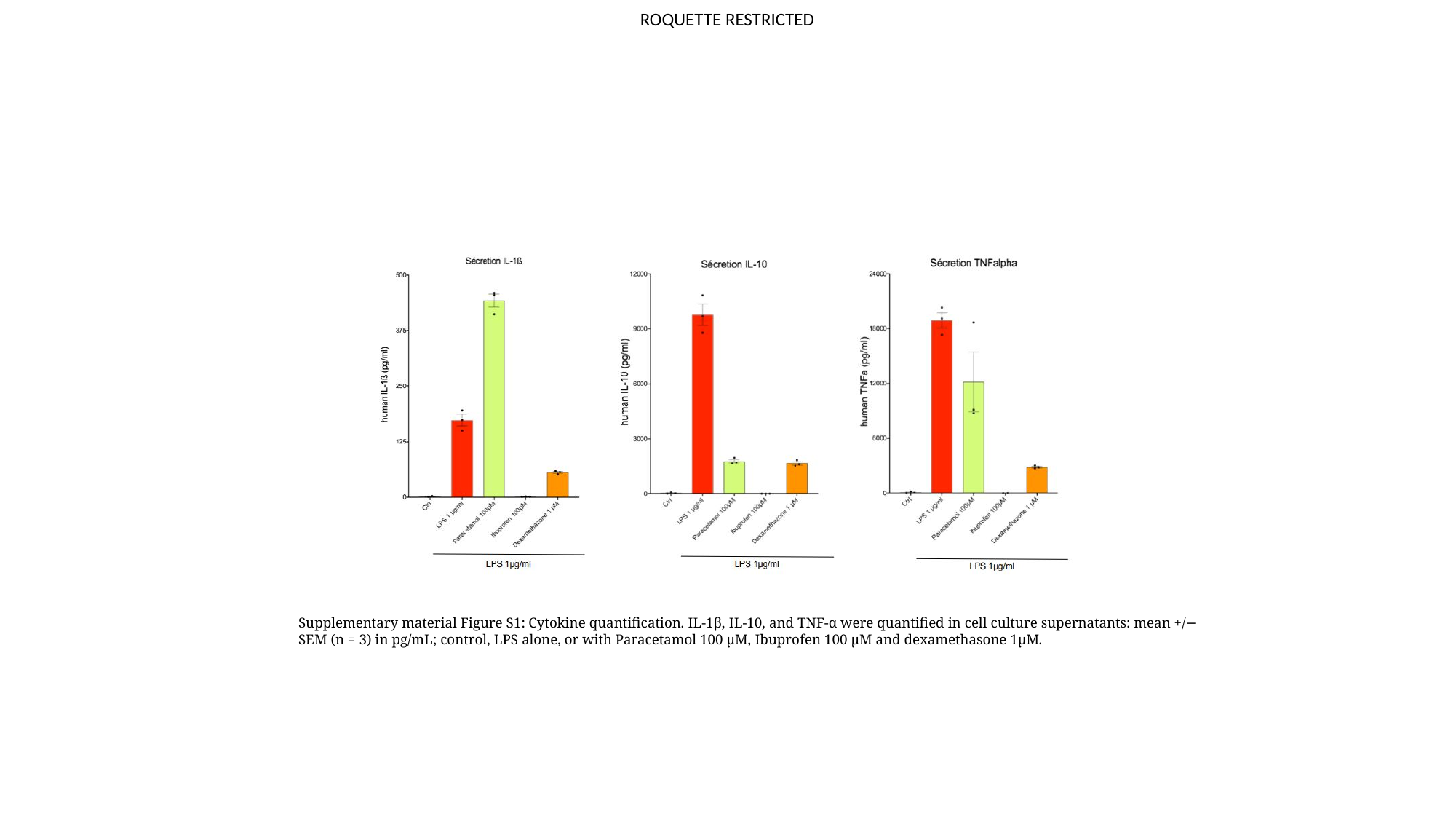

Supplementary material Figure S1: Cytokine quantification. IL-1β, IL-10, and TNF-α were quantified in cell culture supernatants: mean +/− SEM (n = 3) in pg/mL; control, LPS alone, or with Paracetamol 100 µM, Ibuprofen 100 µM and dexamethasone 1µM.
